# Supplementary material for: Patterns of Intron Gain and Loss in Fungi
Source: PLoS Biol. 2004 Nov 30;2(12):e422. doi: 10.1371/journal.pbio.0020422 (PMC532390; doi:10.1371/journal.pbio.0020422)
Supplement: Table S1 — Also available at http://genes.mit.edu/NielsenEtAl/. (4.3 MB ZIP). [file pbio.0020422.st001.zip › NielsenEtAl/html/212.html]

AN9436.1.NCU01625.1.MG03132.1.FG04295.1


```
 CLUSTAL W (1.82) Multiple Sequence Alignments - Introns Inserted


Sequence 1: NCU01625.1	821 aa
Sequence 2: FG04295.1	761 aa
Sequence 3: MG03132.1	805 aa
Sequence 4: AN9436.1	797 aa
Alignment Length: 847 aa
Number Identitical Residues: 538 aa
Alignment Score (without introns) 23744


MG03132.1 	---------------MLFYID2DLPVIFPYPRIYP-~---------E1TLDVGGNCVLEM
NCU01625.1	MTRSRSWSVKNQITNLPANNS~DLPVLFPYPRIYPE1QYAYMVDLKK1TLDAGGNCVLEM
FG04295.1 	---------------------~--------------~--------MQ1TLDAGGHCVLEM
AN9436.1  	---------------MKFFID2DLPVLFPYPRIYPE~QYAYMCDLKK~TLDAGGHCVLEM
          	                    . . .   . .   .. . :   .  : ***.**:*****

MG03132.1 	PSGTGKTATLLSLIVAYQQHYPEHRKLIYCSR1TMSEIEKALAELKNLMKYRAGELGKEE
NCU01625.1	PSGTGKTVTLLSLIVAYQQYYPEHRKLIYCSR~TMSEIEKALVELRALMKFRAERLGHEE
FG04295.1 	PSGTGKTVSLLSLIVAYQQYMPEKRKLIYCSR1TMSEIEKALVELKSLMKYRAEELGYEE
AN9436.1  	PSGTGKTVTLLSLIVAYQQHYPEHRKLIYCSR~TMSEIEKALAELRELMKFRSEQLGYTE
          	*******.:**********: **:******** *********.**: ***:*: .**  *

MG03132.1 	DFRGLGLTSRKNLCLHPSVKREKSGAIVDARCRGLTAGFVKEKKEKGEDVELCVYHD0NL
NCU01625.1	EFRGLGLTSRKNLCLHPSVKREKSGAIVDARCRSLTAGFVKEKREKGENVETCVYHD~NL
FG04295.1 	EFRGLGLTSRKNLCLHPSVKREKSGAIVDARCRSLTAGFVKEKKERGENVDVCVYHD0NL
AN9436.1  	DFRALGLTSRKNLCLHPSVKREKSGTVVDARCRSLTAGFVKEKKEKGEDVELCVYHE0NL
          	:**.*********************::******.*********:*:**:*: ****: **

MG03132.1 	DLLEPHNLIPNGVWTLEGLLRYGEEHKQCPYFTSRRM0------MQYCNVIIYSYHYLLD
NCU01625.1	DLLEPHNLIPNGIWTLDGLMRYGEEKKQCPYFTARRM0------MQHCNVIIYSYHYLLD
FG04295.1 	DLLEPHNLIPNGVWSFDDIIRYGEEQKQCPYFTARRM0------MQYCNVVIFSYHYLLD
AN9436.1  	DLLEPHNLVPPGVFTLDGLLNYGEQHKQCPYFSARRM0PLTPVQMPYCNVIIYSYHYLLD
          	********:* *:::::.::.***::******::*** . :. .* :***:*:*******

MG03132.1 	PKIAERVSKELSRDSIIVFDEAHNIDNVCIESLSTDITEDSLRKASRGAMNLEKRINEMR
NCU01625.1	PKIAERVSKDLSKDCIVVFDEAHNIDNVCIESLSTDITQDSLMRATRGAQNLEAKISEMK
FG04295.1 	PKIAERVSKDFSKDCIVVFDEAHNIDNVCIESLSTDITEDSLRKATRGAQNLENKISQMR
AN9436.1  	PKIAERVSKEFSKDCIVVFDEAHNIDNVCIESLSIDITEDSLRKATRGANNLERKIQDVK
          	*********::*:*.*:***************** ***:*** :*:*** *** :*.:::

MG03132.1 	ETDQNQLEDEYRKLVEGLREADEARQEDAFMSNPV1LPDDLLKEAVPGNIRRAEHFVAFL
NCU01625.1	DSDQEKLQSEYEKLVEGLKGLDEGRQEDAFMANPT~LPDDLLKEAVPGNIRRAEHFVAFL
FG04295.1 	DTDQEQLQNEYQKLVQGLRDADEARQEDAFMANPA1LPEDLLKEAVPGNIRRAEHFIAFL
AN9436.1  	SSDAEKLNNEYMKLVEGLREAEQARDEDQFISNPV~LPDDLLKEAVPGNIRRAEHFVAFL
          	.:* ::*:.** ***:**:  ::.*:** *::**. **:*****************:***

MG03132.1 	KRFIEYLKASNSLSSQIYVYFQLTLLIQTRMKVLEVISETPPSFLAHLKEYTFIEKKPLR
NCU01625.1	RRFIEYLK--------------------TRMKVRQVISETPPSFLAHLKEYTFIEKKPLR
FG04295.1 	KRFIEYLK--------------------TRMKVRQVISETPPSFLAHLREHTFIEKKPLR
AN9436.1  	KRFIEYLK--------------------TRMKVTHTISETPLSFLNHVKELTFIERKPLR
          	:*******                    ***** ..***** *** *::* ****:****

MG03132.1 	FCAERLTSLVRTLELTNIEDFQPLQEVATFATLVATYDKGFLLLLEPYESEKSQIPNPVL
NCU01625.1	FCAERLTSLVRTLELTNIEDYRPLQEVATFATLVATYEKGFLLILEPYESDTAKTPNPVL
FG04295.1 	FCAERLTSLVRTLELTNIEDYQPLQEVATFATLVATYEKGFLLILEPFESDTAEVPNPVL
AN9436.1  	FCAERLTSLVRTLELINIEDYQPLQEVATFATLVSTYDKGFLLILEPFESEAATVPNPVL
          	*************** ****::************:**:*****:***:**: :  *****

MG03132.1 	HFTCLDAAIAIKPVFERFKTVIITSGTLSPLEMYPKMLGFDAVVQESYAMTLARRSFLPM
NCU01625.1	HFTCLDAAIAIKPVFDRFSSVIITSGTISPLEMYPKMLNFETVVQESYAMTLARRSFLPM
FG04295.1 	HFTCLDAAIAIKPVFDRFYSVIITSGTISPLEIYPKMLDFQTVIQESYAMTLARRSFMPM
AN9436.1  	HFTCLDAAIAIKPVFDRFSSVVITSGTLSPLEMYPKMLGFTTVMQESYSMTLARRSFLPM
          	***************:** :*:*****:****:*****.* :*:****:********:**

MG03132.1 	IVTKGSDQASISSSFAVRNDPAVVRNYGQLLTEFAKITPDGMVVFFPSYFYMESIISMWQ
NCU01625.1	IVTRGSDQASISTSFQVRNEPSVVRNYGNLLTEFARITPDGMVVFFPSYLYMESIISMWQ
FG04295.1 	IVTRGSDQASVSTSFQVRNEPSVVRNYGNLLTEFAKITPDGMVVFFPSYLYMESIISMWQ
AN9436.1  	IVTRGSDQAQISSSFQIRNDPGVVRNYGNIVLEFSRITPDGVVVFFPSYLYMESIISMWQ
          	***:*****.:*:** :**:*.******::: **::*****:*******:**********

MG03132.1 	GMKILDEIWKYKLILVETPDAQETAVALETYRTACCNGTGAIMLCVARGKVSEGIDFDHQ
NCU01625.1	GMGILDEVWKHKLILVETPDAQETSLALETYRTACCNGRGAILLCVARGKVSEGIDFDHQ
FG04295.1 	GMGVLDEVWKYKLILVETPDAQETSLALETYRTACCNGRGAVLLCVARGKVSEGIDFDHQ
AN9436.1  	GMGILDSIWNYKLILVETPDAQESSLALETYRTACCNGRGALLFCVARGKVSEGIDFDHH
          	** :**.:*::************:::************ **:::***************:

MG03132.1 	YGRTVLCIGVPFQYTESLILKARLEFLRETYRIRENDFLSFDAMRHAAQCLGRVLRGKDD
NCU01625.1	YGRTVLCIGVPFQYTESRILKARLEFLRETYRIRENDFLSFDAMRHAAQCLGRVLRGKDD
FG04295.1 	YGRTVLCIGVPFQYTESRILKARLQFLRETYRIKENDFLSFDAMRHAAQCLGRVLRGKDD
AN9436.1  	YGRAVLCIGVPFQYTESRILKARLEFLRENYRIRENDFLSFDAMRHAAQCLGRVLRGKDD
          	***:************* ******:****.***:**************************

MG03132.1 	YGIMVLADRRFPKKRPQLPKWINQALAESDVNMSTDMAVSAARKFLKSMAQPFRPEDQDG
NCU01625.1	YGVMVLADRRFMKKRAQLPKWINQAFLEANTNLSTDMAVGNARAFLKQMAQPFSAKDQEG
FG04295.1 	YGIMVLADRRFQKKRTQLPKWINQGLQESDVNLSTDMAVSSARRFLRTMAQPFRAKDQEG
AN9436.1  	YGIMVLADRRFQKKRNQLPKWISQALLESETNLSTDMAVATAKNFLRTMAQPFKAKDQEG
          	**:******** *** ******.*.: *::.*:******. *: **: ***** .:**:*

MG03132.1 	VSRWTIEDLKKHQAKVENDMIRELQ----AAQGKATNGQAVTVRVIEEDED----YEMDD
NCU01625.1	VSIWGMEDLKRHQEKMAEDMIVELKTAREEEERERKRREEEERRRFEEERDRVSDYEMDE
FG04295.1 	ISTWGYKDLMEHKEKMDLERIKEL-----EEEAQRPQPAAQENN-FEYDDD-----ELDQ
AN9436.1  	ISTWSLADLERHRQKQIQEQERLQR----ETFAQGDRTNGSRNG----AGD-----EFDE
          	:* *   ** .*: *   :              :  .             *     *:*:

MG03132.1 	AAEKDMMQLDA---
NCU01625.1	DDEAEMMALDARIG
FG04295.1 	D----MMEMDGF--
AN9436.1  	GIDEDLMMLDAQ--
          	  . .:* :*.
```
